# Plant epigenomics—deciphering the mechanisms of epigenetic inheritance and plasticity in plants

**DOI:** 10.1186/s13059-017-1260-9

**Published:** 2017-07-06

**Authors:** Claudia Köhler, Nathan Springer

**Affiliations:** 1 Uppsala BioCenter, Department of Plant Biology and Forest Genetics, The Swedish University of Agricultural Sciences and Linnean Center for Plant Biology, Almas Allé 5, SE-750 07 Uppsala, Sweden; 2 College of Biological Sciences, University of Minnesota, 306 Biological Sciences, 1445 Gortner Avenue, St Paul, MN 55108 USA

It is an exciting time to study plant epigenetics. Technological advances are providing unprecedented opportunities to monitor chromatin modifications, gene expression, and genome structure. Many classical epigenetic phenomena (transposable element inactivation, imprinting, paramutation, transgene silencing, and co-suppression) were first documented in plants. Combined with classical genetic studies, newly available sequencing technologies are facilitating the study of these and other epigenetic phenomena at a level of detail that was unthinkable only a few years ago. Studies of epigenetics in plants are of great importance. Plants are heavily dependent upon changes in gene expression in order to respond to environmental stimuli, and chromatin-based regulation of gene expression is likely crucial for these responses. Furthermore, the level of chromatin ‘resetting’ during sexual reproduction appears to be lower in plants in comparison with animal species [1, 2], potentially allowing inheritance of epimutations acquired during plant life. In addition, many plant species can propagate asexually and produce vegetative clones, providing opportunities for mitotic inheritance of epigenetic states leading to important traits. This issue of *Genome Biology* highlights exciting progress in many areas of plant epigenetics and epigenomics.

DNA methylation is a well-studied chromatin modification in animals and plants that can be stably inherited, both following cell divisions and, to some extent, across generations. DNA methylation can be monitored at high resolution by using sodium bisulfite treatment of DNA, followed by next-generation sequencing. Cytosines in different sequence contexts (CG, CHG, and CHH (where H is any base other than G)) and at different types of loci in plant genomes can be targeted by DNA methylation. This modification has likely evolved as a mechanism to silence transposons, which are ‘genomic parasites’ invading the genome of their hosts. The vast majority of transposons are highly methylated and are likely a primary target for epigenetic silencing. However, the repetitive nature of transposons and the fact that they generate large insertion/deletion polymorphisms among genotypes has led to difficulties in monitoring the link between transposon polymorphism and DNA methylation variation. Daron and Slotkin describe a new tool to study the interactions between transposon methylation and transposon insertions using whole-genome bisulfite sequencing datasets [3]. This type of analysis is expected to be very useful in documenting the role of genetic and epigenetic variation in DNA methylation among individuals of the same species.

The RNA-dependent DNA methylation (RdDM) pathway is crucial for maintenance of CHH methylation and requires the plant-specific RNA polymerases IV and V (Pol IV and V, respectively). Pol IV generates precursor transcripts of 24-nt small RNAs (sRNAs) that target scaffold transcripts from Pol V by sequence complementarity and recruit the domains rearranged methyltransferase 2 [4]. A rather unexpected link between RdDM and the chromatin remodeling factor PICKLE (PKL) is revealed by Zhang and colleagues, who report that PKL is required for the accumulation of transcripts generated by Pol V and for the positioning of Pol V-stabilized nucleosomes at a subset of RdDM target loci [5]. These findings link nucleosome positioning with the initiation of RdDM, consistent with the previously proposed role of SWI/SNF chromatin remodeling complexes in establishing positioned nucleosomes on specific loci primed for RdDM [6]. It is well established that PKL regulates plant development and, in particular, regulates the access of Polycomb-group proteins to its targets [7]. Likewise, SWI/SNF complexes have well-described roles in plant development [7], extended by the study of Benhamed and colleagues in this issue showing that the SWI/SNF complex core subunit BAF60 regulates access of the Phytochrome Interacting Factor 4 (PIF4) to nucleosome-free regions [8]. The dual functional role of chromatin-remodeling factors in regulating plant development and RdDM suggests that both processes are more closely connected than is widely appreciated.

One enigmatic type of methylation—gene body methylation (gbM)—refers to the moderate levels of CG methylation found within the exons of transcribed genes, and correlates with intermediate levels of expression [9]. Now, three articles in this issue [10–12] provide new insights into DNA sequence features and chromatin factors that might play important roles in gene body methylation [9]. Picard and Gehring use the offspring of a cross between two *Arabidopsis* accessions—with varying levels of methylation at many loci—to elucidate the inheritance patterns of gbM [10]. They find little evidence for a role of gbM in gene expression variation among *Arabidopsis* ecotypes and also highlight the factors that control stable and unstable inheritance of DNA methylation. Schmitz and colleagues document the evolution of chromomethylase genes in a wide variety of plant species and link differences in this gene family to differences in gbM among species [11]. Finally, the work from Berger and colleagues highlights the potential role of histone variants in gene body methylation in *Arabidopsis* [12]. *Arabidopsis* plants with reduced levels of histone variant H3.3 exhibit reduced gbM and altered patterns of histone H1, suggesting an antagonistic relationship between both histones.

While stable silencing of transposons is mediated by DNA methylation, silencing of defined genes during cell differentiation is mediated by Polycomb-group proteins that assemble into the major complexes Polycomb Repressive Complex 1 (PRC1) and PRC2. Both PRC complexes have enzymatic activity, with PRC1 applying monoubiquitination on histone H2A, and PRC2 applying trimethylation marks on histone H3 (H3K27me3). Based on models established for mammals and flies, it has long been assumed that PRC1 is recruited by its ability to bind to H3K27me3 and thus depends on PRC2 activity. Nevertheless, recent data show that PRC1 recruitment can occur independently of PRC2 and, in contrast to the initial model, PRC1 is able to recruit PRC2 [13]. Turck and colleagues in this issue provide evidence that PRC1-dependent recruitment of PRC2 is evolutionarily conserved and also occurs in plants, furthering our understanding of gene regulation mediated by Polycomb-group proteins [14].

The role of histone modifications in mediating the response of plants to changing environmental conditions is highlighted by two articles in this issue. Trimethylation of histone H3 on lysine 36 (H3K36me3) has previously been shown to mark the borders of exons and to connect chromatin structure and RNA processing [15]. Immink and colleagues in this issue reveal that H3K36me3 is required for high-ambient temperature-controlled flowering by affecting alternative splicing of functional transcripts, linking the temperature response to histone modifications [16]. Data by Hirt and colleagues in this issue show that microbe-specific molecules trigger mitogen-activated protein kinases to phosphorylate the plant-specific histone deacetylase HD2B and regulate its function, thereby establishing a connection between pathogen-responsive protein kinase signaling and the chromatin response [17].

Whether specific epigenetic changes have accompanied plant domestication is an important question of potential relevance for plant breeding. Chen and colleagues identify differentially methylated genes between wild and cultivated cotton that have potentially contributed to domestication traits, including flowering-time and seed dormancy, opening new opportunities for breeding of polyploid crops by epigenetic engineering [18].

Collectively, this special issue on plant epigenomics provides insights into current research topics in the field of plant epigenetics that have been moved forward by using next-generation sequencing technology. We witness the enormous progress that has been made from the initial discovery of epigenetic phenomena up until now, where we are able to pinpoint epigenetic modifications throughout the genome. Next, we look forward to seeing these discoveries being translated into practical applications of benefit for breeders and consumers.
